# The Source of Wrinkles

**Published:** 1890-02

**Authors:** 


### THE SOURCE OF WRINKLES.

The general impression about wrinkles is that they are caused by worry, but the truth is that most of them come from laughing. To know how to laugh is just as important as to know when to do it. If you laugh with the sides of your face the skin will work loose in time, and wrinkles will form in exact accordance with the kind of a laugh you have. The man who always wears a smirk will have a series of semi-circular wrinkles covering his cheeks. A gambler, who is accustomed to suppressing his feelings, generally has a deep line running from each side of his nose to the upper corner of his mouth, which in time extends to the chin, forming the shape of a half moon. A cadaverous person is usually marked with two wrinkles, one on the jaw and the other under the eye, meeting at right angles at the cheek bones. The scholar's wrinkle forms on his brow, while a schemer's wrinkles comes around his eyes, and looks like spokes of a wheel.

